# Therapeutic Strategies in the Management of COVID-19

**DOI:** 10.3389/fmolb.2020.636738

**Published:** 2021-02-04

**Authors:** Rajashri R. Naik, Ashok K. Shakya

**Affiliations:** ^1^ Department of Biopharmaceutics and Clinical Pharmacy, Pharmacological and Diagnostic Research Center, Al-Ahliyya Amman University, Amman, Jordan; ^2^ Department of Pharmaceutical Sciences, Pharmacological and Diagnostic Research Center, Al-Ahliyya Amman University, Amman, Jordan

**Keywords:** COVID-19, hydroxychlorquine, chloroquine, lopinavir/ritonavir, remdesivir, favipiravir, arbidol, ivermectin

## Abstract

Since December 2019, SARS-CoV-2 (COVID-19), novel corona virus has caused pandemic globally, with rise in the number of cases and death of the patients. Vast majority of the countries that are dealing with rise in the active cases and death of patients suffering from novel corona viruses COVID-19 are trying to content the virus by isolating the patients and treating them with the approved antiviral that have been previously used in treating SARS, MERS, and drugs that are used to treat other viral infections. Some of these are under clinical trials. At present there are no therapeutically effective antiviral present and there are no vaccines or drugs available that are clinically approved for treating the corona virus. The current strategy is to re-purpose the available drugs or antiviral that can minimise or reduce the burden of the health care emergencies. In this article the reuse of antiviral, US-FDA approved drugs, plant based therapeutic, anti-malarial, anti-parasitic, anti–HIV drugs and the traditional medicines that are being currently used in treating the symptoms of COVID–19 patients is discussed emphasis is also given on the treatment using monoclonal antibodies. The present article provides the therapeutic strategies that will qualify as one of the best available treatment for the better management of the COVID–19 patients in order to achieve medical benefits.

## Introduction

Viruses are obligate parasites which mean they require host cells to reproduce and increase their number; they cannot replicate on their own. These viruses contain single or double stranded DNA or RNA as their genetic material. Viruses are infectious non-cellular particles (López-García and Moreira, 2012). These are mostly contagious and are known to infect almost all the living cells. These are tiny particles that cannot be observed under a normal microscope due to their sub-micron sizes that range from 150–200 nm. A virion is a functional virus that contains genetic material enclosed in an envelope, and the surface proteins help the virus infect the host cell and enter the host cell (Lodish et al., 2000).

Coronaviruses (CoVs) can infect mammals and other species. They are the largest group of viruses and its name is derived from Latin meaning “crown” or “wreath.” This is about the appearance of the surface that has a lipid membrane embedded with club-shaped projections that give the image or appearance of a stellar corona (Masters, 2006). These viruses belong to the order Nidovirales, family Coronaviridae*,* and subfamily Orthocoronavirinae*.* The subfamily Orthocoroanavirinae is divided further into four subgroups which include alpha, beta, gamma, and delta CoVs. The viruses belonging to the order Nidovirales have a 5′ capped, enveloped, single-stranded positive sense RNA virus with an unusual large RNA genome (∼30 kb) with sizes ranging from 80–120 nm (Belouzard et al., 2012). Generally, these viruses have spikes protruding from the surface and replicate in a unique way (Weiss and Navas-Martin, 2005). The RNA of this virus acts like messenger RNA and synthesizes two long proteins that are needed by the virus to replicate when it infects the cell. The SARS-CoV-2 genome encodes many proteins like the nucleocapsid protein, spike (S) protein, envelope (E) protein, and membrane protein (M) that are required for the replication into the host cell. It also codes for the coronavirus main protease that plays an important role in the gene expression and cleaves polyprotein-related proteins (Graham et al., 2008; Chang et al., 2020).

Since late December 2019 the world has being experiencing the problem of increasing cases of COVID-19. The SARS infection first appeared in Wuhan in the Hubei province of China. As of December 2020, there are around 78.7 million active cases and 1.73 million deaths across the world. It has emerged on six continents and has increased exponentially in some countries like the US, Brazil, and India. It was at first called a novel coronavirus belonging to Coronaviridae, beta coronavirus genera and subgenus sarbecovirus and this novel coronavirus was officially named by the International Committee on Taxonomy of Viruses (ICTV) as SARS-CoV-2 (Graham et al., 2008) due to its similarities with the SARS (severe acute respiratory syndrome) outbreak in 2003 (Graham et al., 2008). The World Health Organization (WHO) announced it as COVID-19 on 11 Feb 2020 and declared a public health emergency on 30 Jan 2020.

SARS-CoV-2 is an airborne virus highly contagious with an ability to transfer. As the current evidence suggests, SARS-CoV-2 is transmitted through respiratory mucus or saliva droplets (with droplet size > 5–10 µm in diameter) either from direct contact with an infected person coughing, sneezing, or talking from a distance of 1 meter or less or as droplet nuclei (particle < 5 µm in diameter) that remain active for a considerably long period of time and can spread to other people within 1 m of distance. Indirectly, the virus can spread through contact with surfaces that have been exposed to the virus which may make its way through the nose, mouth, or conjunctiva (Chang et al., 2020; Prasad and Prasad, 2020). It may be suggested that the face, nose, and eyes are often ignored by people as the portal entry for the COVID-19 infection. New evidence on the virus suggests that gastrointestinal symptoms are due to the presence of the viral RNA of this virus or the live infectious particles of the virus found in the feces suggesting that fecal-oral transmission may be one of the other possible routes of transmission of this virus (Coronaviridae Study Group of the International Committee on Taxonomy of, 2020). It is quite intriguing to know that there is no vertical transmission of this virus as evident from the two case studies conducted by (Hindson, 2020; WHO, 2020).

A large number of patients infected with COVID-19 are either asymptomatic or show mild symptoms and recover from the sickness. The clinical interpretations are fever, dry cough, and shortness of breath (Chen et al., 2020b) There are reports on patients experiencing anosmia, dysgeusia, and diarrhea (Fan et al., 2020). However, the mortality rate and the chance for the need of ICU care increases in elderly patients over 60 years of age and patients with underlying conditions like hypertension and diabetes (Shah et al., 2020).

There are no approved vaccines or any specific antiviral therapies available for COVID-19 (Lau et al., 2005). Therefore, the only preventive options left are to give supportive, symptomatic care, social distancing, and quarantine (Coleman and Frieman, 2014). It is a well-known fact that it takes months and years to develop a vaccine from scratch. Hence, researchers are constantly looking for options to manage or treat patients with drugs that are approved and used in treating other diseases like SARS, MERS, or the influenza virus within acceptable safety limits. Some anti-malarial and antiviral treatments have shown some promising results in the management of COVID-19. Some of the drugs that are approved for human disease have shown an antiviral effect by either blocking the viral enzyme which prevents its entry, or by blocking the replication of the genome that is necessary for the formation of the virus particle (Zumla et al., 2016).

In the present article, we have summarized the current therapeutic strategies, vaccines, and other diagnostic options that are being used in the management of COVID-19 and to shed some light on the intervention measures that could be taken to control the outbreak. This article provides information on the therapeutic strategies that will help to address the issues of the pandemic and control them.

## Pharmacotherapeutic agents used in treatment and management of COVID-19

The following pharmacotherapeutic agents are used currently in the treatment and management of COVID-19. Most of the drugs are repurposed agents that are used for the treatment of the disease (Table 1).

**TABLE 1:** Clinical trial and their therapeutic benefits for the management of COVID-19.

Sn.	Name of drug (s)/treatment	Type of study	Therapeutic Benefit	Adverse/side reactions	Reference
1	Hydroxychloroquine, azithromycin	Open label non-randomized clinical trial	Patient treated with hydroxychloroquine and azithromycin were virologically cured compared with patients treated with hydroxychloroquine alone and control group.	1 patient stopped treatment due to nausea on day 3	Gautret et al. (2020a)
2	Chloroquine	Multileft clinical trials	State council of China stated that Chloroquine phosphate was found to be markedly effective in controlling the deteriorating condition of COVID-19 patients in many of the clinical trial Centers in China in Feb 2020	—	Gao et al. (2020)
3	Hydroxychloroquine/Azithromycin	Sngle arm observational study	HCQ and azithromycin (3) showed 100% recovery of the COVID-19 patients and tested negative within 6 days of treatment	—	Raoult (2020)
4	Hydroxychloroquine	Guidance	81% of the patients showed improvement in the condition of pneumonia in the HCQ group compared to 55% in the control group	—	Chen et al. (2020f)
5	Hydroxychloroquine with azithromycin	In-vivo	Rapid clearance of virus. The therapeutic effect was more significant in combination than HCQ alone	—	Megarbane and Scherrmann (2020)
6	Hydroxychloroquine with azithromycin	Uncontrolled non-comparative observational study	They observed that no evidence of a strong antiviral activity or clinical benefit of the combination of hydroxychloroquine and azithromycin for the treatment of hospitalized patients with severe COVID-19		Molina et al. (2020)
7	Hydroxychloroquine with azithromycin	Uncontrolled non-comparative observational study	Clinical improvement, Out of 80 patients, one died, one was admitted to the ICU, and the rest were negative on day 7.		Gautret et al. (2020b)
8	Hydroxychloroquine	Randomized open-label controlled trial: pilot study	Both group (HCQ /supportive care and supportive care) performed equally.	one patient suffered from severe symptoms	Chen et al. (2020c)
9	Hydroxychloroquine with azithromycin	Retrospective cohort study	Mortality was high in HCQ group, followed by combination with azithromycin, and no HCQ. Increased overall mortality in HCQ group.		Magagnoli et al. (2020)
10	Remdesivir	Case Report	Improvement of patient condition	—	Holshue et al. (2020)
11	Remdesivir	Inconclusive study using investigational antiviral drug	12 Patient were given treatment till improvement	GIT symptoms	Kujawski et al. (2020)
12	Remdesivir	Double-blind, randomized, placebo-controlled trial of intravenous remdesivir	The recovery time is reduced from 15 to 11 days as compared to control group	—	Beigel et al. (2020)
13	Favipiravir	Randomized Clinical Trial	The viral clearance time was reduced to 4 days compared to 11 days.	Adverse effects caused by Favipiravir are mild and manageable	Chen et al. (2020a)
14	Lopinavir and ritonavir	Retrospective study	Lopinavir showed beneficial effect in COVID-19	—	Yan et al. (2020)
15	Arbidol/Lopinavir and ritonavir	A retrospective cohort study	In patients with COVID-19, the apparent favorable clinical response with arbidol and LPV/r supports further LPV/r only.		Deng et al. (2020)
16	Lopinavir/ritonavir, arbidol	Retrospective single-centre study	Lopinavir/ritonavir, arbidol were used in a small number of patients. Corticosteriods were used only in case of emergency situation. 215 patients discharged, 22 admitted to ICU,	8 patients developed ARDS, 2 patients died	Chen et al. (2020d)
17	Arbidol	Clinical trial underway	No data	GIT irritation	Jun et al. (2020)
18	Lopinavir/ritonavir, Recombinant human inteferon-α2b Recombinant cytokine gene derived protein, arbidol and Chinese medicines	Double centre observational study	97.9% patients recovered, Lopinavir/ritonavir 75.9, Recombinant human interferon- α2b:45.4%, Recombinant cytokine gene derived protein:18.9%, arbidol 17.2% and chinese medicine:96.6%	2 patients died	Chen et al. (2020e)
19	Interferon β-1b, Lopinavir/ritonavir, and Ribavirin	Open Labeled Clinical Trial Phase-2	Used along with other drugs like Lopinavir and ritonavir showed improvement in viral shedding	Nausea and diarrhea	Huang et al. (2020), Hung et al. (2020)
20	Baricitinib		Pilot study showed beneficial effect of baricitinib over LPV/r therapy		Mehta et al. (2020)
21	Ivermectin	Icon Study	Patient with Ivermectin showed significantly lower mortality rate than control group	-	Rajter et al. (2020)
22	Ivermectin	Hospital-based matched case-control study	Prophylactic effect on health care worker showed 73% reduction in COVID infection	—	Behera et al. (2020)
23	Convalescent Plasma	Multileft Study	Clinical improvement observed in 5000 patients. Convalescent plasma efficacy is inferior to remdesivir when treating COVID-19 patients. Convalescent plasma may be used as a supportive treatment in COVID-19 patients, but must be given as early as possible from the diagnosis.	More than 1% patients showed adverse side effects	Joyner et al. (2020)
24	Convalescent Plasma	Randomized Clinical Trial	52% patients showed clinical improvement with no difference in mortality rate		Li et al. (2020)
25	Interferon Alfacon-1 Plus Corticosteroids	Non-randomized clinical trial	Better clinical outcome in COVID-19 patients with corticosteroids		Loutfy et al. (2003)
26	Dexamethasone	Several trials	Dexamethasone reduces the mortality in severely ill COVID-19 patients.		Horby et al. (2020)
27	Antiviral, Arbidol, Lopinavir/ritonavir, interferon, Ribavirin, oseltamivir, Fluoroquinolones	Multicentre retrospective cohort study to analyze data and treatment of 60severe cases	34 received IV corticosteroid, 28 received IgG; 50 patients improved, 2 patients discharged, 8 remained in a serious condition	4 patients developed secondary infection received glucocorticoids	Huang et al. (2020)
28	Lopinavir/ritonavir	Case report	Treatment with Lopinavir 200 mg /ritonavir 50 mg; Observations indicate reduction in viral load and improvement of symptoms	complaint of depression, insomnia, suicidal thoughts	Lim et al. (2020)
29	Lopinavir, Interferon-a2b atomization inhalation	Retrospective observational single centre study	Treatment with Lopinavir 400 mg , 7 patients discharged, 3 stopped Lopinavir, 2 deteriorated and 1 patient was hospitalized for longer period	digestive problem and hypokalaemia	Liu et al. (2020a)
30	Lopinavir/ritonavir	Retrospective case series study	5 patients out of 10 were discharged, other still under treatment.	—	Lo et al. (2020)
31	Arbidol, Lopinavir/ritonavir, interferon inhalation immune enhancer	Single-centre retrospective case study	22 patients out of 155 died	not reported	Mo et al. (2020)

### Hydroxychloroquine and Chloroquine

**FIGURE 1:**
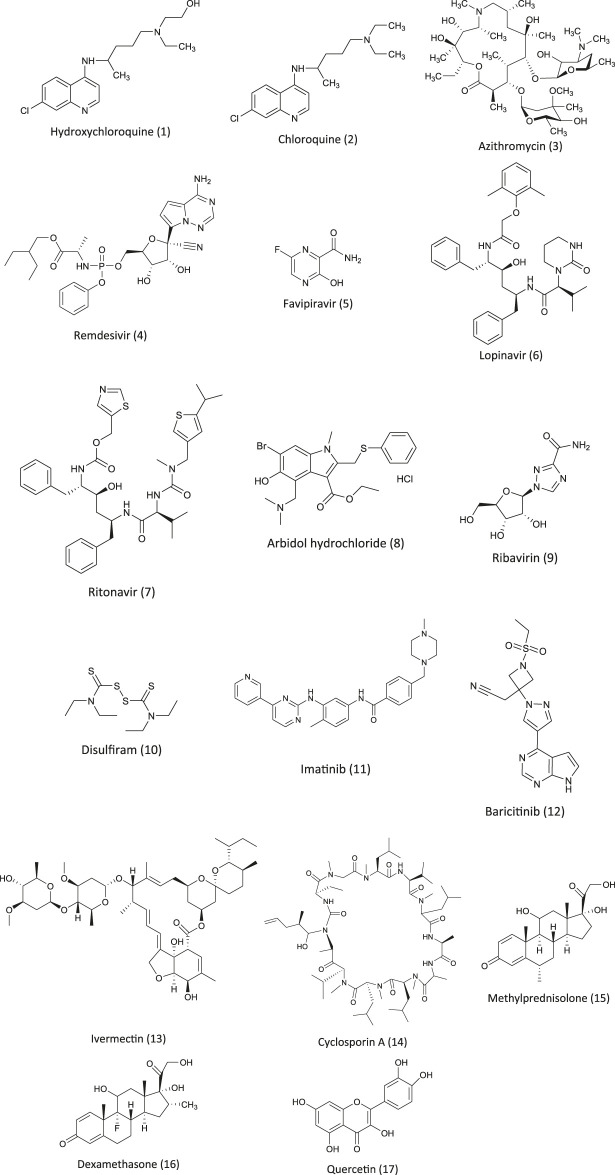
Chemical structures of pharmacotherapeutic agents used in the management of COVID-19 as repurposing agent.

Hydroxychloroquine (Figure 1) and chloroquine (2) have been widely used as antimalarial drugs caused by the plasmodium species. Both chloroquine and hydroxychloroquine are the derivatives of the organic compound that belong to the quinoline group (Goel and Gerriets, 2020). It is one of the active chemical constituents found in the bark of the *Cinchona officinalis* plant. The antimalarial drugs chloroquine and hydroxychloroquine can also be used in treating amebiasis (Knight, 1980), in certain autoimmune diseases like rheumatoid arthritis (Freedman and Steinberg, 1960) and in lupus erythematosus syndrome (Liu et al., 2020b). Chloroquine and its derivative hydroxychloroquine exhibit antimalarial activity by inhibiting the enzyme heme polymerase in the trophozoites resisting the conversion of heme to hemozoin resulting in the accumulation of heme which is toxic to the parasite and kills it (Coronado et al., 2014). These antimalarial agents exert an antiviral effect by multifactorial mechanisms like increasing the pH of the intracellular vacuole and by interfering in the degradation of the protein pathways and modifying the ACE2 receptor glycosylation (Savarino et al., 2006; Sahraei et al., 2020). Angiotensin-converting enzyme 2 (ACE2) is a membrane bound protein and receptor for SARS-CoV. It facilitates its entry into the host cell by binding to the spike (S) protein of the virus resulting in the fusion of the viral and host membrane (Dimitrov, 2003; Li et al., 2003; Simmons et al., 2004). In treating a viral infection like SARS-CoV, it is of utmost importance to prevent the binding of the spike (S) protein of the virus to the ACE 2 by blocking it (Yeung et al., 2006). Chloroquine is being commercialized for its antimalarial activity and used as a drug for autoimmune diseases. It is also known to have a broad spectrum antiviral effect (Yan et al., 2013) and due to its inhibitory effect on ACE2, it is known to be a SARS-CoV inhibitor (Vincent et al., 2005). Chloroquine and hydroxychloroquine have been studied intensively both *in vitro* and in vivo for its antiviral effect on SARS-CoV. Initial *in vitro* studies showed some promising positive results (Gautret et al., 2020a). However, there are no consistent reports available on the clinical trials of these drugs (Molina et al., 2020). CQ seems to be more effective *in vitro* in controlling 2019-nCoV, and it may be considered for the cases of human suffering from COVID-19 (Wang et al., 2020a). A similar inhibitory activity of CQ for SARS-CoV-2 was observed in vero E6 cells at a micromolar level (EC_50_ = 1.13 µM) and was found to inhibit at both the entry and post entry stages in vero E6 cells (Wang et al., 2020a). CQ was found to be effective in inhibiting the aggravation of the virus when compared with the control group in 100 COVID-19 patients. It was also found to reduce the disease and cause negative results among positive COVID-19 patients (Gao et al., 2020). In a press briefing, the state council of China stated that chloroquine phosphate was found to be markedly effective in controlling the deteriorating condition of COVID-19 patients in many of the clinical trial centers in China in Feb 2020 (Gao et al., 2020). The (Guideline for the Prevention, Mar 17, 2020) recommends: for patients aged between 18–65 (adults), a dose of 500 mg twice daily for 7 days; for patients who weigh more than 50 kg, 500 mg twice daily for 2 days; for patients less than 50 kg, 500 mg once daily for 5 days; and is prohibited to patients with cardio problems due to its cardiotoxicity. However, more attention should be paid to the side effects of CQ on patients based on the prior observations made in patients that were treated for other viral diseases and due to its cardiotoxicity. There are several online reports and media reports available regarding the benefits of CQ As of now, many clinical trials are under way in evaluating its effectiveness and its safety in treating COVID-19 patients (NIH, 2020).

Hydroxychloroquine (HCQ) is an antimalarial and immunosuppressive drug. *In vitro* studies found that HCQ was more effective in inhibiting SARS-CoV-2 than CQ (Yao et al., 2020). At present, there are many clinical trials underway on the efficacy of HCQ on COVID-19. As mentioned earlier, the clinical studies are inconsistent. A clinical study done by Dr. Didier Raoult revealed that patients treated with HCQ and azithromycin (3) showed 100% recovery of the COVID-19 patients and tested negative within 6 days of treatment (data not published). Due to its positive *in vitro* results on SARS-CoV-2, it was used on a large scale for COVID-19 patients in many countries including the USA. Other countries like South Korea and China that are affected by the pandemic have issued or underlined the guidelines for the therapeutic use of HCQ in treating COVID-19 patients. The Comprehensive Treatment and Management of COVID-19: Expert Consensus Statement from Shanghai recommends the use of hydroxychloroquine without dosage recommendation. The Peking University developed a PBPK model, and based on the result of this model, they recommended an oral dose for COVID-19 patients (Qu et al., 2020).

This was later confirmed by a clinical trial with 62 patients, where 31 received HCQ in a dose of 400 mg/d, 200 mg twice daily for 4 days while 31 other patients were the control group. After five days, the recovery period for the patients in the HCQ group was shortened and they showed significant recovery from the symptoms of fever and cough; further, it was observed that 81% of the patients showed improvement in the condition of pneumonia in the HCQ group compared to 55% in the control group. It should be mentioned here that the patients who were critically ill were from the control group, which confirms the therapeutic use of HCQ (Qu et al., 2020; Guideline for the Prevention, Mar 17, 2020). In another study, 36 COVID-19 patients were given HCQ with azithromycin. A full and rapid clearance of the virus was observed. The effect was more significant when given in combination with azithromycin then HCQ alone. It is still not clear how azithromycin enhances the efficacy, but it is known to inhibit the virus in severe respiratory infections (Megarbane and Scherrmann, 2020). The results were not similar in a small study of 11 COVID-19 patients conducted by (Molina et al., 2020) using the same combination of HCQ and azithromycin. This study was with a small number of patients, and the control group was from another clinical center, so the study was not a randomized controlled trial. This paper also did not undergo peer review and was published within 24 hours of its submission. In order to confirm its results, an expanded clinical trial with correct protocols should be followed. In another clinical trial, 42 COVID-19 patients were recruited. Of these 42 treated patients, six were excluded from the study (three patients were admitted to ICU, one died, one showed an adverse drug reaction, and one refused to take the medication) (Gautret et al., 2020b). The remaining 36 patients were taken into consideration for the final analysis. Twenty patients were given HCQ 200 mg orally three times daily with six of these patients given azithromycin along with HCQ to enhance the efficacy of HCQ. The patients who refused to take the drug were treated as the control which was from the other hospital. They found that six of the 36 patients showed no symptoms, 22 of the remaining patients complained of a sore throat and the remaining eight patients had signs of pneumonia. The primary goal of the clinical trial was the clearance of the virus, and the secondary was the serial viral load, clinical follow up, and the adverse effect of the drug. Both drugs have long QT intervals and increase the chances of tip torsion arrhythmia which may lead to death. Hence, a thorough evaluation is required (Malviya, 2020). In another observational study, 80 patients with mild COVID-19 infection were given hydroxychloroquine 200 mg three times daily for ten days along with azithromycin 500 mg orally for one day followed by 250 mg daily for four days (Gautret et al., 2020b). It was observed that out of the 80 patients, one died, one was admitted to the ICU, and the rest showed rapid recovery with a rapid decrease in the viral load in nasopharyngeal which was negative on day 7 with 83% and 93% on day 9, and the respiratory viral culture showed negative results with 97.5% on day 5. The hospital stay was reduced to five days. This study too had limitations as this was an observational study without any comparative group. And, the beneficial effects of this study cannot be evaluated as the study was on patients with mild symptoms. A pilot study was conducted in which they recruited 30 patients who were randomized into two groups of 15 each with one group receiving 400 mg of HCQ daily along with supportive care and the other group receiving only supportive care (Chen et al., 2020c). They observed a negative clearance of the virus on day 7 by RT-PCR and one patient suffered from severe symptoms. However, it was observed that there was no significant difference between the two groups in the clearance of the virus; with respect to clinical and radiological parameters, both groups performed equally the same. They concluded that the study was good, but a larger group should be included to evaluate the effectiveness of HCQ on COVID-19 patients. This paper was published in a Chinese journal and it is not clear whether it was peer reviewed. In a retrospective study of 368 patients hospitalized due to COVID-19 (Magagnoli et al., 2020), the patients were divided into 3 groups with the first group consisting of 97 patients who were given HCQ alone, the second group consisting of 113 patients who were given HCQ and azithromycin, and the third group consisting of 158 with no HCQ. In this study, only two primary result data were taken into consideration, one the death rate and the other the use of mechanical ventilation. The mortality rate was 27.8%, 22.1%, and 11.4% in HCQ alone, HCQ + azithromycin and no HCQ, respectively. The use of mechanical ventilation was 13.3%, 6.9%, and 14.1% in the same three groups, respectively. It was observed that the death rate was higher in the HCQ group then no HCQ group, and there was no significant difference in the use of ventilation in all three groups. The authors observed no beneficial effect of HCQ alone or with azithromycin or no HCQ on ventilation. An increase in the mortality rate was observed in the HCQ alone treated group. This study raises the credibility of the efficacy of HCQ for COVID-19. More randomized clinical control trials are required to address the issue. There are a large number of trials going on or reported (Borba et al., 2020; Mahevas et al., 2020; Molina et al., 2020; Tang et al., 2020) but none of them provide clear-cut data on whether to use or not use HCQ in treating COVID-19.

Even the results on the prophylactic role of HCQ for health care workers (Chatterjee et al., 2020) and individuals with a high risk of exposure to COVID-19 (Boulware et al., 2020) showed contradictory results. Another placebo controlled trial testing HCQ on post exposure for prophylaxis effect (Boulware et al., 2020) showed no significant difference in the occurrence of COVID-19 like symptoms between participants that received HCQ [49 of 414 (11.8%)] and those who received a placebo [58 of 407 (14.3%)]. It was also observed that the side effects were more in the HCQ participants than the placebo ones (40.1% in HCQ participants and 16.8% in placebo participants) but the side effects were without any adverse reaction. From their study, they concluded that HCQ could not prevent the occurrence of COVID-19 after exposure for high or moderate risk individuals. The study had certain limitations as most of the participants including the health care workers were unable to assess testing and the diagnosis was based on the symptoms that were more similar to the disease. The Indian Council on Medical Research (ICMR) studied the pre-prophylaxis effect of HCQ on health care workers (Boulware et al., 2020; Chatterjee et al., 2020). They concluded that there was a significant decline in the odds of getting infected by the health care workers who were given a maintenance dose of HCQ. A dose response relationship was seen between the rate of exposure and reduction in the infection. These two drugs are immunomodulators and are not immune suppressants (WHO, 2017). Hence, these drugs have no adverse effect related to an infection or cancer (Lazarus et al., 2006; Schrezenmeier and Dorner, 2020). The most common adverse side effects associated with these two drugs are nausea, vomiting, and abdominal discomfort (Srinivasa et al., 2017), but CQ and HCQ may cause cardiotoxicity, myopathy, and retinopathy (Costedoat-Chalumeau et al., 2007; Abdel-Hamid et al., 2008; Chatre et al., 2018; Jorge et al., 2018) and have long QTc intervals (Costedoat-Chalumeau et al., 2007; WHO, 2017; Chatre et al., 2018). Though the margin of safety for HCQ is significant (WHO, 2017; Ursing et al., 2020), in seriously ill COVID-19 patients, it causes cardiac problems and death related to it (Guastalegname and Vallone, 2020) which may be due to the high dose (Borba et al., 2020) in combination with azithromycin which increases the QT intervals (Malviya, 2020) and due to the patients’ underlying conditions (WHO, 2017; Chatre et al., 2018).

In the present scenario, the role of CQ or HCQ in the management of COVID-19 is a dynamic phenomenon, and as the results of the clinical trials become available, their role may change. As of now, there are many clinical trials underway, and researchers are awaiting the results. The clinical trials should include a large number of participants with different age groups and different underlying conditions. They should also include patients who are asymptomatic, mild symptomatic, and severely ill. These studies should have varying drug doses. *In vitro* studies have shown that these two drugs have antiviral properties, but the main question is how far these *in vitro* studies can be transformed or utilized in clinical studies (Rathi et al., 2020; Taccone et al., 2020). So far the clinical trials are not convincing enough for the use of HCQ in the management of COVID-19 patients . Depending on the results of the clinical trials available at this time, the experts are against the use of HCQ in the management of COVID-19 patients. Despite all of these contradictory opinions, there has been widespread advocacy to use HCQ mainly due to the fear that surrounded around COVID-19 and media/social forces rather than scientific facts (Cohen, 2020). Question arises on the safety of these both drugs, CQ and HCQ, and there is a thin line when it comes to the safety. Hence, if used indiscriminately and without proper supervision, they may lead to severe side effects mainly related to a craniological effect. If the drug is used under supervision as a prophylactic agent and as mentioned or advocated by ICMR as safe and encouraging (Chatterjee et al., 2020) with good positive results, the drugs would be a blessing in blocking the virus. Otherwise, it may be an antimalarial that can undergo clinical trials.

### Remdesivir

Remdesivir (4) was developed as an antiviral agent by an American biotechnology company called Gilead Science to treat the Ebola virus. Remdesivir is a prodrug that is metabolized to GS 441524, a nucleoside analogue, which inhibits the RNA dependent enzyme RNA polymerase (Gordon et al., 2020). This affects the function of the RNA polymerase that inhibits the viral enzyme endonuclease involved in the proofreading activity resulting in a decrease in the production of viral RNA (Agostini et al., 2018). Remdesivir exhibits a broad spectrum of antiviral activity and has shown encouraging results against SARS-CoV and MERS-CoV infections. Due to its inhibitory activity, many physicians have recommended the use of remdesivir in countries like the United States, Europe, and Japan (Bloomberglaw.com, 2020). In patients suffering from SARS-CoV, remdesivir has not been recommended by drug regulatory authorities. An *in vitro* clinical isolate of SARS-CoV showed that remdesivir exhibited significant inhibitory activity. There are studies available on the efficacy of remdesivir. There are many clinical trials underway to test the efficacy of remdesivir on COVID-19. In China, two clinical trials were started to study the efficacy of remdesivir on SARS-CoV-2. At Capital Medical University, a randomized quadruple-blind placebo controlled phase III clinical trial was registered to determine the efficacy of remdesivir on patients showing mild and moderate symptoms of a COVID-19 infection (NCT04252664, (ClinicalTrials.gov., 2020a)). A second trial on patients with advanced SARS-CoV-2 was registered at the same center (ClinicalTrials.gov., 2020b). In both the trials, on day one patients were administered with 200 mg of remdesivir, followed by 100 mg for 9 days. In both trials, the main aim was to note the time taken for the clinical trials, time taken to bring the fever to normal, saturation of oxygen, respiratory rate, and relief of cough within 72 hours.

Similarly, almost at the same time, the first COVID-19 case was reported in the United States (Holshue et al., 2020). The patient was suffering from a fever for four days which was later confirmed as COVID-19. When his condition worsened on the seventh day, he was given remdesivir by IV and his condition improved with no adverse side effect (Holshue et al., 2020). The clinical state of the patient improved the next day, but it was confounding to analyze the impact or the efficacy of remdesivir because the patient received simultaneous treatments of acetaminophen, ibuprofen, guaifenesin, vancomycin, cefepime, and supplemental oxygen. Later during the same period, 12 patients were confirmed with SARS-CoV-2 (Kujawski et al., 2020). Of the 12 patients, seven were hospitalized. Upon the worsening of their condition, three patients were given remdesivir (on compassionate grounds). The treatment was given for 4–10 days. Each patient received 200 mg of remdesivir IV on day one followed by 100 mg/day. It was observed that all the patients showed transient gastrointestinal symptoms like nausea, vomiting, gastroparesis, or rectal bleeding. Even though all the patient reported the symptoms, the treatment was continued until improvement (Kujawski et al., 2020). As the study was a small size and did not have any control group, it is hard to summarize the effect of remdesivir or the safety of this drug on COVID-19 patients. Funded by the National Institute of Allergies and Infectious Disease (NIAID), the NIH developed a study on the existing Chinese clinical trial in consultation with the WHO. The study was a double blind, randomized placebo controlled phase III trial which was aimed to evaluate the safety and efficacy of remdesivir in comparison with a placebo (ACTT-1 ClinicalTrials.gov number, NCT04280705) (clinicaltrials.gov, 2020). At present, the patients are being enrolled and the severity of the patients’ conditions is noted based on an eight-point ordinal scale along with secondary outcomes. An estimate of 75 clinical trials is expected to participate in this study across the United States and is expected for the primary completion by April 2023.

Professor Cao Bin at the China-Japan Friendship Hospital in a clinical study suggested that remdesivir does not have any significant beneficial role or has no antiviral effect on patients suffering for severe SARS-CoV-2 (Wang et al., 2020c). However, there are several studies on the efficacy of remdesivir in COVID-19 patients. The results of the clinical trials from around the world suggest that remdesivir is known to reduce the symptoms and the mortality rate in patients on ventilation in intensive care units (Grein et al., 2020). Meanwhile the results of several clinical trials conducted in Chicago suggest that remdesivir is known to have a beneficial effect in early COVID-19 patients due to reduction in lung damage (Williamson et al., 2020). The results published on the use of remdesivir in severe COVID-19 patients suggest that 68% of severely affected patients have reduced symptoms and 13% mortality. Although the results are encouraging, it needs to be confirmed in a randomized, placebo controlled clinical trial on COVID-19 patients (Grein et al., 2020). The efficacy and safety of remdesivir has not been confirmed yet. It is available in several ongoing clinical trials for adults and non-pregnant patients, but on compassionate grounds the use of remdesivir is limited to pregnant women and individuals <18 years of age suffering from severe COVID-19. Although there is no contradiction indicated in the clinical trials, people with liver and kidney impairment must be treated or taken care of with caution (Guo et al., 2020). Under the compassionate use program, patients with confirmed COVID-19 have been given remdesivir in places like the US, Europe, and Japan (Amirian and Levy, 2020). Some of the initial studies in China have shown that remdesivir has no role in the clinical improvement of patients (Wang et al., 2020c). Results of the clinical trials on the adaptive COVID-19 Trial (ACTT-1) showed the recovery time was reduced significantly in the treatment group compared to the control (11 days compared to 15 days with a ratio rate of recovery 1.32; 95% confidence interval [CI], 1.12 to 1.55; *p* < 0.001) (Beigel et al., 2020). Although there are no results to validate that remdesivir decreases SARS-CoV-2 or RNA viral loads or reduces the mortality rate (Wang et al., 2020c), earlier positive results encourage the use of remdesivir. Furthermore, the results of the trials are still pending to validate the present results.

Based on the findings, the United States Food and Drug Administration (US-FDA) has approved the emergency use of remdesivir in hospitalized COVID-19 patients. With no other drug being approved by the US-FDA for use in the treatment of SARS-CoV-2, remdesivir may lessen the mortality, morbidity, and the burden of the global pandemic (FDA-News, 2020).

### Favipiravir

Favipiravir (T-705) (5) is a purine nucleic acid analog. In RNA viruses, favipiravir is known to inhibit the RNA dependent RNA polymerase (RdRp). After phosphoribosylation and phosphorylation, it forms favipiravir-RT which terminates the elongation of the RNA strand by binding to the RdRp (Sangawa et al., 2013). Due to its antiviral activity, favipiravir was licensed to treat influenza in Japan in 2014 (Hayden and Shindo, 2019). It is also known to be effective against several other viruses like Ebola, influenza, norovirus, chikungunya, and enterovirus (De Clercq, 2019). Favipiravir is shown to have teratogenic and embryotoxic effects and should be avoided during pregnancy and lactation (Nagata et al., 2015). *In vitro* studies on E6 vero cells suggest that favipiravir was able to interfere with the function of SARS-CoV–2 (EC50 = 61.88 Μm) (Wang et al., 2020a). The results encouraged more than 10 clinical trials which are underway or registered and patients are being recruited for the study. The initial results from one of the trials (ChiCTR2000029600) showed some promising results in terms of virus clearance which was short (4 days vs. 11 days) and the chest image (91.43% vs. 62.22%) when compared to the control group (with lopinavir and ritonavir). In 45 cases, the adverse reaction in comparison with the control group was lower (Cai et al., 2020). Another randomized clinical trial at the hospital at Wuhan University (ChiCTR2000030254) concluded that the patients treated with favipiravir (for 120 patients) showed a significant recovery rate (7 days) as compared to the control which was treated with umifenovir (for 120 patients). They concluded that the group treated with favipiravir were significantly better than the control group (Chen et al., 2020a). Encouraged by the positive results from the clinical trials and the availability of the drug, experts have recommended that the drug be included in the guidelines of National Health Commission of the People’s Republic of China.

### Lopinavir/Ritonavir

Lopinavir and ritonavir (LPV/r) (6, 7) is an FDA approved oral combination drug that is used in the treatment of HIV-1. As an antiretroviral protease inhibitor, lopinavir is metabolized in the liver where ritonavir, a CYP3A4 inhibitor, is also a protease inhibitor and in combination with lopinavir, it is used to boost its antiviral activity and its bioavailability (De Clercq, 2009). It was used in the outbreak of SARS in 2003 due to its antiviral activity. Therefore, when there was a SARS-CoV-2 pandemic, LPV/r was considered as one of the best options to treat SARS-CoV-2. In vero E6 cells, lopinavir is known to inhibit the replication of SARS-CoV-2 with EC50 of 26.63 mM (Choy et al., 2020). In another *in vitro* study, Kang et al. showed that LPV/r significantly inhibited the activity of SARS-CoV-2 with a concentration of 7/1.75 mg/ml (Kang et al., 2020). In one of the clinical studies on hospitalized COVID-19 patients, the analysis showed that the early administration of LPV/r resulted in a short virus shedding (Yan et al., 2020). However, the results were not similar in the randomized clinical trial which showed that LPV/r had no beneficial effect on COVID-19 patients. In another randomized clinical trial in China, the combination drug was given twice daily for two weeks to adult patients with severe COVID-19. It was observed that there was no significant beneficial effect to the patients when compared to the control (standard care) group (Cao et al., 2020). In the same study, due to an adverse effect, only 14% of the patients could complete the treatment. Similar lack of benefit in the patients was observed in the clinical trial conducted in UK (unpublished data)(Randomized Evaluation of COVID-19 Therapy). The study resulted in terminating the recruitment of patients for the treatment. The treatment of LPV/r has adverse side effects like anorexia, nausea, abdominal discomfort, diarrhea, or acute gastritis, along with the risk of liver damage, inflammation of pancreas, more severe skin eruptions, and also the interaction of the drug with the CYP3A inhibition was observed in the clinical trials. These adverse reactions raised concerns related to the high dose and long term use of LPV/r to improve the clinical condition of the patients (Cao et al., 2020). In context to the above-mentioned adverse effects, there are serious concerns related to kidney injury, and secondary infection was less in those who were not receiving treatment. There are very limited data available on the use of lopinavir/ritonavir in treating COVID-19 patients. Further trials involving patients with severe COVID-19 symptoms and large control clinical trials are needed, and patients with underlying conditions need to be involved to study the role of lopinavir/ritonavir.

### Arbidol

Arbidol (8), an indole-derivative molecule, has been approved as a prophylactic and to treat influenza and other respiratory infections caused by a virus (Blaising et al., 2014). Arbidol is being used in Russia and China against an upper respiratory tract infection due to the influenza virus A and B. Arbidol has inhibitory activity against diseases such as hepatitis B and C (Boriskin et al., 2008). Antiviral activity against SARS pathogens has been reported, and *in vitro* antiviral activity of the derivative arbidol mesylate (derivative of arbidol) showed a five times higher reduction in the reproduction of SARS then arbidol (Khamitov et al., 2008). There is a report available on the *in vitro* study on the efficacy of arbidol against COVID-19 (Lu, 2020). Arbidol exerts antiviral activity by inhibiting hemagglutinins which is a protein on the membrane of the virus which binds to the sialic acid receptor on human cells and makes its entry into the human cell by endocytosis and prevents the virus from being infective. It also induces the production of interferon and exhibits broad antiviral activity (Blaising et al., 2014). (Deng et al., 2020) observed that in SARS-CoV-2 patients taking a combination drug of arbidol and LPV/r, the negative rate increased in 7 and 14 days and the CT scan of the chest improved when compared to the oral LPV/r combination. However, it has to be noted that no improvement in the symptoms due to LPV/r or arbidol was observed. The clinical trial at the Shanghai Public Health Clinical Center observed more adverse reaction related to the gastro intestine (Jun et al., 2020). These contradictory results may be due to a small trial size and the timing of medication. A clinical trial is underway in China on post exposure prophylaxis in a population with a high risk of COVID-19. In this study, health care staffs were included (ChiCTR2000029592). The outcome of this result may throw light on the efficacy of arbidol on the early treatment and prevention of COVID-19.

### Ribavirin

Ribavirin (9), a guanosine nucleoside, is a synthetic antiviral drug. It exhibits its antiviral activity by inhibiting the enzyme inosine monophosphate dehydrogenase and shutdowns the progress of the replication of RNA and DNA viruses that leads to the destruction of the genome RNA. Currently, this drug has been approved and used in the treatment of chronic hepatitis C (Koren et al., 2003). It is known to inhibit viral replication in MERS-CoV when given to patients in combination with other antiviral drugs. It was observed that ribavirin when administered in combination with interferon alfa-2a, 70% of the patients who received this combination were alive for 14 days when compared to 29% in the control. In *in vitro* studies, ribavirin exhibited antiviral activity against the respiratory syncytial virus and influenza and para influenza virus, and due to its antiviral activity, ribavirin has been recommended by the US-FDA to treat patients with respiratory syncytial virus, where it had no antiviral activity against SARS-CoV (Koren et al., 2003). With respect to SARS-CoV-2, there is new evidence that suggests that ribavirin may decrease the replication of the virus by inhibiting PLpro (Wu et al., 2020). Recently in an open label randomized clinical trial (phase II), patients treated with a ribavirin, LPV/r, and interferon-1b combination had viral shedding more quickly when compared to patients with LPV/r (Fujii et al., 2004; Hung et al., 2020). As this study lacked a placebo group and critically ill patients, and it lacked ribavirin and a IFN-ß-1b monotherapy group, it was difficult to assess the use of this drug alone and it needs to be further studied. It has to be noted that ribavirin has more adverse reactions, such as hemolytic anemia (Fujii et al., 2004), hypocalcemia, and hypomagnesemia (Koren et al., 2003; Tai, 2007). It is being currently investigated in combination with other antivirals for therapy, but there are no reports available on the use of ribavirin alone in the treatment of COVID-19.

### Disulfiram

Approved by the US-FDA (United States Food and Drug Administration), Disulfiram (10) is used in aversion therapy for alcohol. It inhibits the enzyme aldehyde dehydrogenase in the liver. The *in vitro* study has shown that disulfiram inhibits PLpro papain–like protease in SARS-CoV and MERS-CoV (Lin et al., 2018). *In vitro* studies have shown that GRL0617, a targeted PLpro inhibitor, blocked the replication in SARS-CoV (Ratia et al., 2008). This compound is in its preclinical study, but it may be considered in the development of an anti-coronavirus drug. So far there are no clinical data available on the efficacy of disulfiram in COVID-19 treatments (McCreary and Pogue, 2020), but there is theoretical evidence that supports the repurposing of disulfiram.

### Imatinib

Imatinib (11), also known as Gleevec, is an inhibitor of oral tyrosine kinase that has antiviral activity against Ebola and poxviruses (Coleman et al., 2016). In SARS and MERS-CoV, Abelson tyrosine kinase 2 (ABl2) is required for replication, and imatinib targets this ABl2. Imatinib inhibits MERS-CoV and SARS-CoV infections in *in-vitro* studies and with significantly low cellular toxicity (Dyall et al., 2014). There are no clinical data available at present and some are yet to be published, but the result of the *in vitro* studies suggest that a clinical trial or study would be beneficial.

### Baricitinib

Baricitinib (12) is used in the treatment of rheumatoid arthritis. It inhibits Janus kinase (JAK), binds to the adaptor associated kinase-1(AAK1) enzyme that is involved in the clathrin-mediated endocytosis, and also stops the cytokine storm (Russell et al., 2020). Based on the computational studies, baricitinib was identified as one of the promising candidates in the treatment of SARS-CoV-2. In a pilot study, they compared baricitinib and LPV/r with the standard therapy given to COVID-19 patients. This study showed that baricitinib decreased the viral entry and the cytokine storm (Mehta et al., 2020). It also showed significant improvement in fever, dyspnea, and hypoxia (Cantini et al., 2020). This drug seems to be one of the promising antiviral protease inhibitors which may be explored further for SARS-CoV-2.

### Ivermectin

Discovered in 1975, Ivermectin (13) came into use in 1981 (Vercruysse and Rew, 2002; Mehlhorn, 2008). It is approved by FDA to treat, prevent and control the river blindness (onchocerciasis) that is common in population. Ivermectin is also used in the treatment of Strongyloidosis, enterobiasis, Trichuris trichura, Loa Loa, Scabies, human lice, malaria. Under *in–vitro* conditions Ivermectin is known to exhibits broad spectrum of antiviral activity against large number of viruses (Mastrangelo et al., 2012; Lundberg et al., 2013; Azeem et al., 2015; Gotz et al., 2016). As mentioned above COVID–19 is a ssRNA virus that is similar to SARS coronavirus (SARS–CoV). Recent *in-vitro* studies on the antiviral activity of Ivermectin against SARS CoV –2 revealed that it can inhibit the viral replication. It reduced the virus load by 5000 fold in vero hSLAM cells submerged with single dose of Ivermectin for period of 48 hour, however increase in exposure period up to 72 hour did not show any effect in the reduction of the viral load. In addition, there was no side effect of this drug at any given point of time (Choudhary and Sharma, 2020). How it exhibits its antiviral activity is not known, it probably exhibits its antiviral activity same way has it does on other different viruses. It inhibits the import of host and viral proteins into the nucleus. Integrase protein of the virus and importin IMP/1 heterodimer is necessary for the IN nuclear import which enhances the infection. During infection majority of the RNA viruses rely on the IMP/1, Ivermectin increases its antiviral potentials by inhibiting the import (Caly et al., 2020; Choudhary and Sharma, 2020). Study on the clinical efficacy of ivermectin was studied by (Rajter et al., 2020) patients hospitalized in four board health hospital in Florida was studied a total of 280 patients was involved (from march 15 to 11 may 2020). Out of 280 patients, 173 patients were treated with ivermectin and 107 without, most patients in both the group received HCQ or azithromycin or both of them. The results showed that the patients treated with ivermectin had significantly lower mortality rate especially in patients with ventilator support and who required inspired oxygen in comparison to control (15.0% vs 25.2%). But this study has short comings this finding must be confirmed by randomized clinical trial. In another study carried out in India on the association between ivermectin prophylaxis and infection of COVID-19 among health care works. A reduction of 73 % in COVID-19 infection was observed in health care works for the following one month. In this study the health care workers were given two–dose of ivermectin prophylaxis at dose of 300 μg/kg with a gap of 72 hours (Behera et al., 2020). This study was not peer reviewed and further clinical trials are required so that it can be used in large scale or to be used for clinical trial. In another retrospective study conducted on hospitalized patients receiving Ivermectin in Spain showed no improvement (Camprubi et al., 2020). They observe that there was no clinical improvement and microbiological outcomes in the severe COVID-19 patients who received 200 μg/kg in comparison to similar group of patients who did not receive ivermectin, It must be noted that the ivermectin treatment was given later in the later stage of the disease or infection. This study has some limitations, randomized clinical trials are necessary to evaluate the high doses. In another randomized clinical trial involving patients with mild to moderate symptoms and who tested positive for COVID–19 was studied (Chowdhury et al., 2020) in Bangladesh Chakoria Upazilla Health Complex. In this study the patients were divided into two group, group A received single dose of 200 µgm/kg + Doxycycline 100 mg BID for 10 days and Group B received 400 mg of HCQ on 1st day and subsequent 9 days they received 200 mg + Azithromycin 500 g for 5 days. Time taken for negative PCR test and full symptomatic recovery was measured in both the groups. Patients in group A (Ivermectin + Doxycline ) showed more promising trend towards recovery then group B. It was observed that the patients in group A showed negative result at a mean day of 8.93 days and full symptomatic recovery at 5.93 days and 55.10 % were symptom free by 5th day. Whereas group B showed negative result at mean of 6.99 days and were symptom free by 9.33 days. These results were not statistically significant between the difference in time for negative PCR and between symptom free and was not peer reviewed. Although the results are promising but more test results are awaited to be used as one of the therapeutic agents.

### Immunomodulators

#### Convalescent Plasma

Passive immunity through antibodies can be used in the treatment of SARS-CoV-2 patients. This is done by using convalescent plasma from recovered patients. The convalescent plasma from infected patients contain antibodies that give immunity to the patients either by binding to the infectious particle-like virus and neutralizing it or through other pathways mediated by antibodies such as antibody-dependent cellular cytotoxicity (Casadevall and Pirofski, 2020) by phagocytosis and complement activation. This therapy has shown encouraging results in other infectious diseases like measles, poliomyelitis, mumps, and influenza (Casadevall and Pirofski, 2020). In 2003 during the SARS-CoV-1 outbreak, convalescent plasma therapy was used to evaluate its efficiency. It resulted in significantly higher 22-day discharge in treated patients when compared to control groups (Cheng et al., 2005), but there are other studies that were inconclusive (Stockman et al., 2006). In South Korea during the MERS outbreak, two of the three patients who received this convalescent plasma were able to neutralize the antibody activity (Ko et al., 2018). Based on the promising outcome of the previous studies or experience, the US-FDA facilitated the use of convalescent plasma on compassionate grounds in critically ill COVID-19 patients (USFDA, 2020). In the case of emerging pandemics where vaccines and antiviral drugs are unavailable, the WHO has authorized the use of convalescent plasma. The study on the efficacy and safety of convalescent plasma was conducted on COVID-19 patients in United States. The preliminary results were encouraging with respect to clinical improvement as 36% of the patients’ demonstrated improvement in 7 days and 76% of the patients improved or were discharged from the hospital. Also, there were no adverse effects reported by the patients, which makes it a safe therapy for severely ill COVID-19 patients (Salazar et al., 2020). Similar results were observed in a study involving 5,000 patients, and < 1% of the adverse effects were observed during the first four days post-infusion with 14.9% mortality (seven-day incidence) (Joyner et al., 2020). In another study involving 39 severely ill COVID-19 patients, Liu et al. found that patients who got convalescent plasma had an improved survival rate in non-intubated patients than in intubated patients (Liu et al., 2020c). In an open label randomized clinical trial involving 103 severely ill COVID-19 patients given convalescent plasma, 52% of the patients showed clinical improvement in 28 days when compared to the control group with 43%. It was noted that there was no difference in the mortality rate in both groups (28 days), but there was a higher negative PCR conversion rate in the treatment group (87.2%) than the control group (37.5%) (Li et al., 2020). This study was terminated due to a decrease in COVID-19 cases. In another study with five patients, (Shen et al., 2020) reported that there were clinical improvements in the patients. However, the efficacy of the treatment could not be assessed due to the small size of the study, but these encouraging results prompt the investigator to study more rigorously the efficacy of convalescent plasma as a possible treatment. A number of clinical trials are underway, and others have been submitted to the US-FDA to test the efficacy of convalescent plasma. These studies may throw some light on the efficacy of the treatment.

#### Interferons

Protein molecules released by the host cell in response to viral infection heighten the immune system of the host and combat the virus. Interferon alfacon-1 is a synthetic type 1 interferon with a 166 amino acid sequence synthesized by genetic engineering (Melian and Plosker, 2001). Interferon alfacon-1 is an antiviral and anticancer agent. It has a therapeutic effect on leukemia, melanoma, HIV/AIDS related Kaposi’s sarcoma, and hepatitis C (Schlossberg and Samuel, 2017). It was found to be effective in SARS-CoV and was tested for COVID-19. In a non-randomized trial, COVID-19 patients were treated with interferon in combination with corticosteroids. Patients treated with interferon alfacon-1 and steroids had a better clinical outcome than patients treated with steroids alone without interferon alfacon-1 (Loutfy et al., 2003). There are *in vitro* studies to show the inhibitory activity of interferon alfacon-1 on SARS-CoV-2. The result of interferon-beta-1b in a combination therapy has been encouraging and clinical trials are underway to evaluate the use of type-1 interferon as an adjunctive therapy.

#### Cyclosporine A

Cyclosporine A (14) is an immunosuppressant that has been widely used in autoimmune disorders and transplantations. The *in vitro* studies have shown that cyclosporine A inhibits SARS and other coronaviruses replication. The mechanism of antiviral activity is not known, but it might be due to the inhibition of cyclophilin protein pathways including SARS-CoV. It may be beneficial for COVID-19 patients, but its use is limited due to its toxic effect. There are some studies where a small number of kidney transplant patients who changed to or continued cyclosporine A during COVID-19 treatment showed no harmful effects (Molyvdas and Matalon, 2020).

### Corticosteroids

#### Methylprednisolone

Methylprednisolone (15) is an immunosuppressive and anti-inflammatory agent approved by the US-FDA. Due to its anti-inflammatory activity at low doses, corticosteroids like methylprednisolone has been tested for a variety of viral infections, but the clinical benefits remain divisive. The use of corticosteroid in RSV infected children had no clinical benefits (Corneli et al., 2007). It also appeared to be harmful in other viral infections like hepatitis and cerebral malaria (McGee and Hirschmann, 2008). A comparative study on the effect of corticosteroids (including methylprednisolone) with a placebo involving 6,548 patients showed that corticosteroids were associated with an increase in mortality (Ni et al., 2019). A systemic review of the use of corticosteroids in SARS revealed that in four studies the use of corticosteroids was harmful including a decrease in viral clearance and drug complications while 25 other studies were inconclusive (Stockman et al., 2006). Many clinical studies on the effect of corticosteroids on viral infections showed that they have no clinical benefits, so the WHO does not recommend the use of corticosteroids in the treatment of viral pneumonia or ARDS. Regarding COVID-19, there is no convincing evidence to show that methylprednisolone has no therapeutics benefits. However, several clinical trials are underway to study the safety and efficacy.

#### Dexamethasone

Dexamethasone (16) is a steroid and due to its anti-inflammatory and immunosuppressant activity, it is being used in diseases like asthma, allergies, and autoimmune diseases like lupus and rheumatoid arthritis (Shrestha et al., 2019; Elkharwili et al., 2020; Wang et al., 2020b). There are no data available on the role played by dexamethasone in the cure of COVID-19 patients, but there is evidence to show that the survival and mortality rate in severely ill COVID-19 patients improves when treated with dexamethasone. Preliminary results of the randomized phase II and III clinical trials showed that dexamethasone reduced deaths in severely ill COVID-19 patients. Out of 11,320 patients, 2,104 were randomized to receive dexamethasone treatment for 10 days, 4,321 received usual cares, and the rest were given standard care. It was found that the mortality rate reduced by one third in patients with ventilation and in patients who required oxygen by one fifth. Dexamethasone had no therapeutic benefit for patients with milder cases and who did not receive oxygen support (Horby et al., 2020) . Currently, there are 14 clinical trials going on, and eight of them are expected to complete their study by 2020. The results may be beneficial in combating this pandemic in severely ill patients.

## Traditional and Alternative Medications

### Chinese Traditional Medicine

During the SARS outbreak in 2003, Chinese traditional medicine was used to treat and prevent the disease (Zhang, 2003). Traditional medicine of China was part of the prevention program to include several Chinese herbal medicine formulas to prevent infection in adults and children. It was observed that the Shufeng Jiedu and Lianhua Qingwen capsules played an important role in the prevention and treatment of influenza A (H1N1). There are also studies to show that Yu ping feng powder has an antiviral, anti-inflammatory, and immunoregulatory effect (Du et al., 2015). A large scale randomized trial found that Yinqiao powder with other heat-clearing formulas could reduce the time of a fever in patients with H1N1 virus infection (Wang et al., 2011). It is also suggested that traditional Chinese medicine may be beneficial in a high risk population that is exposed to COVID-19, such as medical staff, family members, and people who come in contact with COVID-19 patients. However, the efficacy and safety of Chinese traditional formulas on COVID-19 patients needs to be confirmed by clinical trials.

### Indian Traditional Medicine

In India, Neem (*Azadirachta indica* L.) is considered as a traditional plant, and the leaves, seeds, bark, flowers, and roots are used to cure various diseases. The active constituent of neem, like Nimbolide has been explored for its pharmacological properties and is used in treating various diseases such as cancer, diabetes, and inflammatory diseases (Elumalai et al., 2012). In an experimental model, Nimbolide was found to inhibit TNF–α, and suppress the nuclear translocation of p65 NF-κB, HDAC-3, and the cytokine storm, so it may have some beneficial effects in the SARS-CoV-2 infection because of its antiviral activity or directly by controlling the cytokine storm. It may also have clinical significance for inflammation during a viral infection. Some of the other natural products that have been used in various diseases and that show antiviral activity against herpes virus 1 and 2 is Withaferin A found in the medicinal plant *Withania sominifera* from Ashwagandha (Grover et al., 2011). It may have or show encouraging results in COVID-19 treatment.

#### Saikosaponins

Belonging to the triterpenoids group, Saikosaponins are extracted from various plants like *Scrophularia scorodonia*, Heteromorpha spp, and Bupleurum spp and is known to exert antiviral activity against Corona virus 229E by inhibiting the penetration and attachment of the virus (Cheng et al., 2006). Molecular docking of Saikosaponins B4 suggests that this compound may be used as a spike protein inhibitor and could be a possible therapeutic agent in the treatment of COVID-19.

#### Quercetin

Quercetin (17) is a plant flavanol that belongs to polyphenols and is used as a pharmacological agent for inflammation and cancer (Jeong et al., 2009). It has shown antiviral activity by inhibiting viral replication and by inhibiting its entry in the Dengue virus. It also exhibited antiviral activity against SARS-CoV by inhibiting its entry into a host cell. It may have antiviral activity against SARS-CoV-2. It has been approved by the US-FDA to use it as an active ingredient in drugs. All these natural and traditional compounds or ingredients need to be clinically tested against COVID-19 for possible use in this pandemic.

## Monoconal Antibodies

Large number of monoclonal antibodies is being used as therapeutic agents and also for diagnostic purposes. USFDA has approved the use of monoclonal antibodies to treat cancer and autoimmune diseases. Few monoclonal antibodies is being used in the treatment of SARS-CoV-2, to name few Bevacizumad (NCT04305106), Tocilizumab (NCT04317092), Meplazumab (NCT04275245) etc. Tian et al. (2020) reported that the human monoclonal antibody like CR3022 that is specific to SARS-CoV has the ability to bind to the SARS-CoV-2 RBD (KD of 6.3 nM) and the epitope did not overlap with ACE2 binding sites in SARS CoV–2 RBD. Making it an clinically effective candidate for treating SARS CoV-2. In developing a new monoclonal therapeutic agent for treating SARS-CoV-2, scientists target the spike proteins, ACE2 binding sites . Large number of monoclonal antibodies is tested against SARS-CoV and these monoclonal antibodies may be effective against COVID–19 (Tian et al., 2020).

### Tocilizumab

To treat autoimmune diseases like Rheumatoid arthritis and multiple myeloma, tocilizumab is used. This is a human recombinant IL-6 receptor (IL-6R) antibody and IL-6 receptors is involved in the activation of inflammatory and immunological modulators that is responsible in the respiratory collapse that is observed in the patients that are infected with COVID-19. It is also associated major side effects like allergy, liver toxicity and hyperlipidemia (Jones and Ding, 2010). At present, tocilizumab is under phase II clinical trials and is being tested for SARS-CoV-2 (NCT04317092) (Scott, 2017).

### Sarilumab

Sarilumab is another monoclonal antibody and is an IL-6 receptor that is under clinical trials for COVID–19 (NCT04315298). It is used in autoimmune disease like rheumatoid arthritis and suppresses the inflammation mediated by IL–6R. The therapeutic efficacy of the sarilumab along with remdesivir against SARS-CoV-2 patients is being tested under the clinical trials. Further clinical trials may be required to study its potential as the most effective therapeutic agent against SARS-CoV-2 (Pelechas et al., 2019; Lansdowne and Campbell, 2020).

## Vaccines

In any given pandemic there will be a rush to develop vaccines to help the mankind. Vaccine development takes time as the vaccines must not only be protective but also safe. Unlike other drugs that are delivered into sick patients, vaccines are administered into healthy patients and require very high safety margins (Singh and Mehta, 2016). During the COVID-19 pandemic several different institute and Pharmaceutical companies are trying to develop the vaccines. As of August 11, 2020, 28 of these companies have advanced into clinical trials with Moderna, CanSino, the University of Oxford, BioNTech, Sinovac, Sinopharm, Anhui Zhifei Longcom, Inovio, Novavax, Vaxine, Zydus Cadila, Institute of Medical Biology, and the Gamaleya Research Institute having moved beyond their initial safety and immunogenicity studies (Chung et al., 2020). The vaccines are in clinical trial or in market as mentioned in Table 2. The Pfizer-BioNTech COVID-19 vaccine (BNT162b2) has not been approved or licensed by the U.S. Food and Drug Administration (FDA), but has been authorized for emergency use by FDA under an Emergency Use Authorization (EUA) to prevent Coronavirus Disease 2019 (COVID-19) for use in individuals 16 years of age and older. (www.cvdvaccine.com). As per the recent articles published indicate that the efficacy of the Pfizer vaccine was 52% after first dose and 95% after taking second dose (Mahase, 2020). The adverse drug reactions are also mentioned in the article. There were 4 deaths during the clinical trials (in the treatment and placebo group), as per the investigators these cases are not related to the vaccine. More clinical trails are in progress to launch these vaccines in the market. According to the some data the vaccines will be first available to the health care workers and people in need.

**TABLE 2:** List of Front-line companies in the development of Vaccines for COVID-19.

Name of Company/Organization	Name of Vaccine	Approval Status
Pfizer	BNT162b2	Approved in United Kingdom. The vaccine has also been granted emergency approval in Canada and conditional approval in the European Union
Moderna Therapeutics	mRNA-1273	On December 18, the FDA granted emergency approval to Moderna’s COVID-19 vaccine
University of Oxford	ChAdOx1 nCoV-19	On December 8, The Lancet published an interim analysis of four of Oxford’s phase three trials. It showed the vaccine is safe and 70.4-percent effective in preventing COVID-19 after two doses, and 64.1-percent effective after one standard dose
The Gamaleya National Center of Epidemiology and Microbiology	Sputnik V	In August, Russia cleared the Sputnik V vaccine for widespread use and claimed it as the first registered COVID-19 vaccine on the market—before the vaccine’s phase three trials had begun and despite the lack of published evidence at the time
Bharat Biotech	COVAXIN	Not approved for use. Under approval process.
Novavax	NVX-CoV2373	Not approved for use.
Johnson & Johnson	JNJ-78436735	Not approved for use.
Murdoch Children’s Research Institute	Bacillus Calmette-Guerin BRACE trial	Not approved for use.
CanSino Biologics	Ad5-nCoV	Though the company was still technically in phase two of its trial, on June 25, CanSino became the first company to receive limited approval to use its vaccine in people. The Chinese government has approved the vaccine for military use only, for a period of one year.
Vector Institute	EpiVacCorona	On October 14, ussia granted regulatory approval to EpiVacCorona even though the vaccine candidate has not published any results and has not entered phase three of its clinical trials. It is the second vaccine candidate that Russia has approved for use despite a lack of published evidence about its safety and efficacy.
Sinovac	CoronaVac	Approved for limited use in China.
Zydus-Cadila	ZyCoV-D	Not approved for use, expected to launch by March 2021

## Conclusion

COVID-19 is causing a pandemic and there is an urgent therapeutic need to combat it. There is no potential antiviral drug or vaccine available to treat this virus. There is a global race to develop a vaccine, but in the present scenario to minimize the strain patients are given the current available therapeutics that has been used in previous viral infections or viral outbreaks. The patients are given the currently available antiviral depending on the prior antiviral experiences regarding SARS, MERS, influenza, and HIV virus, and these drugs have been evaluated in clinical studies or trials with patients. In this review, we have summarized the use of the current available drugs that have potential antiviral activity, and emphasis was given to the drugs that have circulated as having potential as an antiviral and anticipated to be beneficial in the treatment of COVID-19. However, most of the studies or the clinical trials were inconclusive or inconsistent in concluding the benefits of the repurposing of the available antiviral drugs. Some of the clinical trials are still ongoing and the results are pending which may be beneficial in treating or minimizing the symptoms of the virus. Some of the results are expected to be by the end of the year. Remdesivir proved to be beneficial from the clinical trial and has been recommended by the US-FDA for its use in the treatment of COVID-19 patients. Although some of the available antiviral have encouraging results, care must be taken and monitored clinically for the possible adverse side effects these drugs may pose. It should be kept in mind to monitor the effect of these drugs on critically ill patients or patients with ventilation and the effect it has on patients with mild symptoms or underlying conditions.
